# Compressed composite carbon felt as a negative electrode for a zinc–iron flow battery

**DOI:** 10.1038/s41598-022-25763-5

**Published:** 2022-12-07

**Authors:** Janenipa Saupsor, Jinnawat Sangsawang, Wathanyu Kao-ian, Falko Mahlendorf, Ahmad Azmin Mohamad, Rongrong Cheacharoen, Soorathep Kheawhom, Anongnat Somwangthanaroj

**Affiliations:** 1 Department of Chemical Engineering, Faculty of Engineering, Chulalongkorn University, Bangkok, 10330 Thailand; 2 Department of Energy Technology, University Duisburg-Essen, 47057 Duisburg, Germany; 3 School of Materials and Mineral Resources Engineering, Universiti Sains Malaysia, 14300 Nibong Tebal, Pulau Pinang Malaysia; 4 Metallurgy and Materials Science Research Institute, Chulalongkorn University, Bangkok, 10330 Thailand; 5 Center of Excellence on Advanced Materials for Energy Storage, Chulalongkorn University, Bangkok, 10330 Thailand; 6 Bio-Circular-Green-Economy Technology and Engineering Center (BCGeTEC), Faculty of Engineering, Chulalongkorn University, Bangkok, 10330 Thailand

**Keywords:** Energy storage, Chemical engineering, Materials for energy and catalysis

## Abstract

Flow batteries possess several attractive features including long cycle life, flexible design, ease of scaling up, and high safety. They are considered an excellent choice for large-scale energy storage. Carbon felt (CF) electrodes are commonly used as porous electrodes in flow batteries. In vanadium flow batteries, both active materials and discharge products are in a liquid phase, thus leaving no trace on the electrode surface. However, zinc-based flow batteries involve zinc deposition/dissolution, structure and configuration of the electrode significantly determine stability and performance of the battery. Herein, fabrication of a compressed composite using CF with polyvinylidene fluoride (PVDF) is investigated in a Zn–Fe flow battery (ZFB). Graphene (G) is successfully introduced in order to improve its electrochemical activity towards zinc reactions on the negative side of the ZFB. A compressed composite CF electrode offers more uniform electric field and lower nucleation overpotential (NOP) of zinc than a pristine CF, resulting in higher zinc plating/stripping efficiency. Batteries with modified electrodes are seen to provide lower overpotential. Particularly, the G-PVDF-CF electrode demonstrates maximum discharge capacity of 39.6 mAh cm^−2^ with coulombic efficiency and energy efficiency over 96% and 61%, respectively. Finally, results lead to increased efficiency and cycling stability for flow batteries.

## Introduction

Due to the rising demand for renewable energy sources such as solar and wind, the development of energy conversion and storage systems is of paramount importance; though such sources are unpredictable and sporadic in nature^1–3^. Moreover, storing energy when it produces more than expected is also necessary. Recently, the tremendous attention of energy storage systems (ESSs) has focused on the expansion of flow batteries^4–7^. Flow batteries possess several attractive features including long cycle life, flexible design, ease of scaling up, high safety, low capital cost and independence of energy and power components^8–11^. So far, vanadium redox flow batteries (VRFBs) have been thoroughly investigated, but they still suffer from low energy density and the high price of vanadium^12–14^. Therefore, many researchers are now focused on alternative active materials such as halides (I and Br), sulfides (S), zinc (Zn) and iron (Fe) to develop lower-cost and higher-performance RFBs, as well as environmentally benign and excellent electrochemical kinetics^15–19^.

Zinc (Zn^2+^/Zn^0^)-iron (Fe^3+^/Fe^2+^) couples are promising active species for high energy density flow batteries^20–22^. The aqueous Fe(II/III) redox couple as a cathode material is among the cheapest and safest, and it is seen to have reasonable high voltage, high solubility and fast kinetics. Metallic zinc is regarded as an ideal anode material for aqueous hybrid flow batteries due to its low potential, abundance, nontoxicity, and cost-effectiveness^9,23^.

The electrochemical cell reactions associated with the ZFB in an aqueous electrolyte are given below^24^:

Negative electrode:

1 $${\text{Zn}}^{0} \leftrightarrow {\text{ Zn}}^{{{2} + }} + {\text{ 2e}}^{ - } \quad \left( {\text{E}}_{0} = - 0.76{\text{ V vs. SHE}} \right)$$

Positive electrode:

2 $${\text{Fe}}^{{{3} + }} + {\text{ e}}^{ - } \leftrightarrow {\text{ Fe}}^{{{2} + }} \quad \left( {\text{E}}_{0} = + 0.77{\text{ V vs. SHE}} \right)$$

Overall cell reaction:

3 $${\text{Zn}}^{0} + {\text{ 2Fe}}^{{{3} + }} \leftrightarrow {\text{ Zn}}^{{{2} + }} + {\text{ 2Fe}}^{{{2} + }} \quad \left( {{\text{E}}_{0} = + {1}.53{\text{ V}}} \right)$$

During charging, metallic zinc is electrodeposited onto the surface of a negative electrode while oxidized Fe^3+^ is dissolved in the electrolyte. As its role in providing Zn electrodeposition, a current collector for negative electrode is one of the battery parts that determine performance and stability of the ZFBs^25–28^. Ideally, the current collector for ZFBs should have high surface area, high electronic conductivity, high Zn compatibility and chemically stable in the electrolyte^21^. Due to the corrosive nature of zinc–iron battery’s electrolyte, carbon-based materials are generally implemented. Among them, carbon felt (CF) stands out due to its good electrical conductivity, excellent corrosion resistance, reasonable cost, three-dimensional structure, and wide operating potential^29,30^. Despite of the high conductivity of the carbon fiber in CF, the CF requires the compression due to the large gap between the fibers. Thus, the felt compression is a common method to improve electrode efficiency as fiber density is increased, resulting in additional self-connection of fibers and better contact with the substrate of the electrode^31^. Several studies have attempted to investigate the influence of compressed carbon felt electrodes on the performance of RFBs^32–34^.

The effect of compression of a carbon felt electrode on the performance of a VRFB has been demonstrated by Chang et al.^35^. A thorough evaluation of its electrical, mechanical and morphological properties of the compressed electrode was carried out. This study tuned the percentage of compression by adjusting the height of the spacer between two flat acrylic plates sandwiching the carbon felt in the range of 0–40%. However, the effect of felt compression on charge–discharge performance was not undertaken. Park et al.^36^ further investigated the influence of compressed CF electrodes on the charge–discharge behavior of a VRFB; the battery is seen to perform well due to the increase in discharge time and maximum power of the cell. In this work, the percentages of compression were controlled by stacking pieces of the PVC gaskets. Brown et al.^37^ used gasket thickness to control the compression of felt electrodes. In their investigation of VRFBs, improved electrochemical performance is achieved, demonstrating greater energy efficiency and cycling stability when a compressed electrode is applied.

The compression of carbon felt electrodes plays a crucial role in enhancing the performance of RFBs because such flow batteries depend heavily on cell resistance during stack assembly. However, compressed carbon felt electrodes are solely based on an increase in cell clamping pressure performed on conventional VRFBs. In this work, a study of the characteristics and performance of a hot-compressed polymer-carbon felt composite electrode for the anodic reaction of a ZFB is presented. Because of the poor physicochemical properties of original CFs, polymeric binders such as PVDF are usually applied to enhance their flexibility and strength^38,39^. Furthermore, it is important to improve compressed composite electrodes by incorporating graphene (G) into PVDF-CF to compensate for electrical performance loss caused by polymer filling. Graphene is known to be a promising active material for electrode applications due to its excellent conductivity and large specific surface area^40–42^.

In particular, this study describes a compressed composite CF electrode revealing much improved mechanical and electrochemical performances. The amount of PVDF in the electrode was investigated in order to achieve the best balance of electrical conductivity and strength requirements. The electrochemical reactivity of the modified electrode towards zinc reactions on the negative side of the ZFB was evaluated using cyclic voltammetry (CV), electrochemical impedance spectroscopy (EIS), and Zn plating/stripping efficiency. A ZFB single cell was used to conduct a charge–discharge test to evaluate battery performance. Various characterization methods have been carried out to understand the relationship between the electrode and electrochemical performance of CF before and after modification.

## Experimental section

### Reagents and materials

Commercial CF (thicknesses of 3 mm) was used as the raw material and purchased from AvCarb Material Solutions. A Nafion117 membrane was purchased from FuelCellStore and used after pretreatments by a standard acid boiling procedure. Polyvinyl fluoride (PVDF, Mw ~ 180,000) was obtained from Sigma-Aldrich, and 1-Methyl-2-pyrrolidinone (NMP AR) was purchased from Quality Reagent Chemical (QReC). Both zinc chloride (ZnCl_2_, 97%) and zinc sulfate heptahydrate (ZnSO_4_·7H_2_O, 99%) were purchased from KEMAUS. Graphene powder was obtained from NanoIntegris Technologies, Inc. All chemicals used in the experiments were of analytical grade and used without further purification.

### Electrode preparation

**Figure 1:**
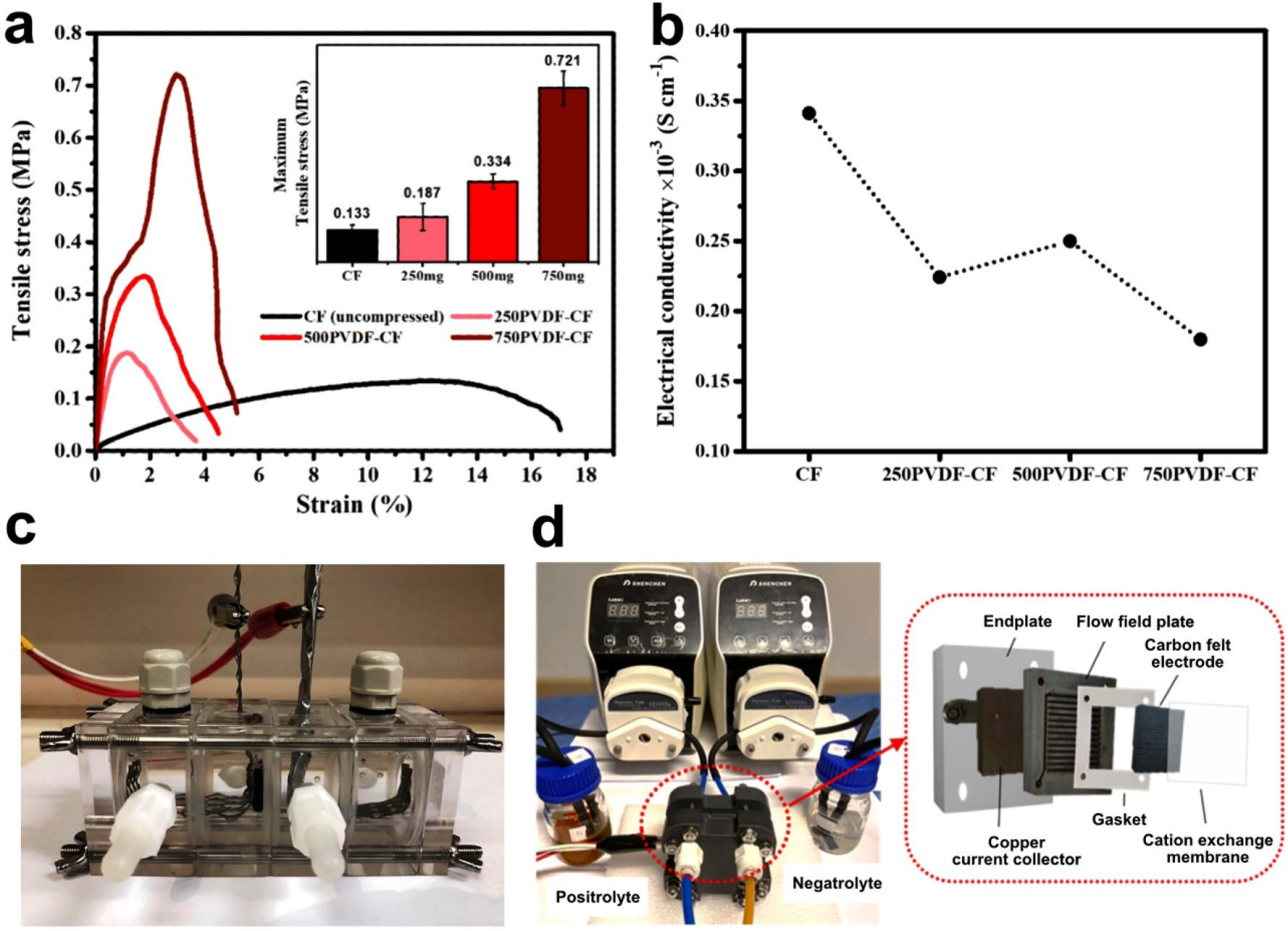
(**a**) Tensile stress–strain curves of CF and PVDF-CF electrodes at different PVDF contents, (**b**) electrical conductivity of CF and PVDF-CF electrodes at different PVDF contents, (**c**) photograph of the two-electrode cell system for Zn plating/stripping tests, and (**d**) photograph and schematic representation of flow cell used in this work.

Carbon felt was cleaned in acetone for 10 min to remove impurities on the surface and then dried in air at room temperature. The original CF was used as the positive electrode, while the original and modified CF were used as the negative electrodes for comparison. As for the modified electrode, CF conductivity is highly influenced by PVDF content. Therefore, different amounts of PVDF (250, 500 and 750 mg) were prepared by dissolving in 25 ml of NMP. Then, the mixture was heated to 55 °C and stirred for 3 h continuously until the solution became clear. Next, the polymer solution was sonicated for 10 min for homogenous slurry. Subsequently, the CF (5 cm × 10 cm) was immersed in the slurry and the slurry with CF was sonicated again for 20 min. The slurry coated samples were dried in an oven at 70 °C overnight to ensure that the NMP was completely removed. Finally, the dried samples were hot-pressed at 175 °C and pressurized at 50 kg cm^−2^ to obtain a sample with 10% compression. The hot-pressed samples were denoted as *x*PVDF-CF where *x* is the amount of PVDF. In Fig. 1a, the tensile strength of each composite electrode is given. The maximum tensile stresses of the PVDF-CF composites proved to be higher than the conventional CF electrode. PVDF composites are seen to increase in the following order: CF (0.13 MPa) < 250PVDF-CF (0.19 MPa) < 500PVDF-CF (0.33 MPa) < 750PVDF-CF (0.72 MPa). The maximum tensile stress was used to determine the optimal amount of PVDF. According to the literature, when maximum stress of the material is greater than 0.26 MPa, the electrode remains in a safe mechanical state^43^. As a result, the 500PVDF-CF and 750PVDF-CF samples appear to be promising candidates. However, as shown in Fig. 1b, higher PVDF content in the CF electrode can cause drawbacks such as lower conductivity. Therefore, 500 mg of PVDF is chosen as an optimal content for the CF electrode. Thus, a graphene modified compressed electrode was prepared for comparison by mixing 25 mg of graphene powder with PVDF slurry (500 mg of PVDF and 25 ml of NMP) before coating and hot-pressing. The sample is denoted by G-PVDF-CF.

### Characterization

The surface morphologies of CF samples were observed via field emission scanning electron microscope (FESEM, FEI Quanta 250 FEG) at an acceleration voltage of 15.0 kV equipped with an energy-dispersive X-ray (EDX) spectroscopy for elemental mapping analysis. X-ray diffraction (XRD) was carried out by XRD system (Bruker AXS, Model D8 Discover) using Cu Kα radiation (λ = 1.5418 Å) at a power of 40 kV × 40 mA. Raman spectra of original and modified electrodes were analyzed by Raman Microscope (Model XploRA PLUS) using a 532 nm exciting wavelength at room temperature. The wettability of the CFs was investigated by contact angle measurement applying a water droplet method using Dataphysics OCA 40.

### Electrochemical measurements

To measure the electrochemical performance in the electrolytes, CV and EIS tests were conducted using a potentiostat and galvanostat device (Squidstat plus, Admiral Instruments, USA) with a conventional three-electrode system. Both an original and modified CF (active area: 1 cm^2^) were used as working electrodes with platinum as the counter electrode, and Ag/AgCl electrode as the reference electrode using a 0.5 M ZnCl_2_ in 3 M KCl solution as the electrolyte. EIS measurements were performed in the frequency range: 0.01 Hz to 1000 kHz with an AC amplitude of 10 mV at the open-circuit potential.

By using the same cell setup as used in CV test (Fig. S1a), the electrochemical active surface area was measured (ECSA) via capacitance measurement within 0.5 M sodium sulfate solution. The CV test, having a potential window of − 0.2 V to 0.75 V and a scan rate of 30 to 100 mV/s, was used to analyze the samples (Fig. S1b–d). The mean current value was calculated from the difference between anodic current and cathodic current at the central potential of the CV curve. Then, the mean current was plotted versus the scan rate (100 mV/s). The slopes of the plot, which are the capacitances of the electrodes, were obtained using linear regression (Fig. S1e). The ECSA values were calculated by dividing the obtained capacitance by the specific capacitance of carbon material (0.02 mF/cm^2^)^44^.

The electrodeposition of Zn on the CF electrode was carried out in a two-electrode system (Fig. 1c). A Zn sheet (1.5 cm × 2 cm) was used as the anode and the prepared CF was used as the substrate. 1 M ZnSO_4_ solution was employed as electrolyte for Zn plating/stripping tests. The gap between Zn sheet and CF was 2 cm. The current density applied for electrodeposition process was 10 mA cm^−2^ for 1 h on BTS-5V50mA (Shenzhen NEWARE, China) battery testing system. As shown in Fig. 1d, the performances of single cells were prepared by stacking the end plate, copper current collector, flow field channel, silicone gasket, CF electrode, and membrane. The active area of the CF electrode for the battery test was 10 cm^2^, and Nafion117 was prepared as the separator. 45 ml of 0.5 M ZnCl_2_ in 3 M KCl and 0.25 M FeCl_3_ in 3 M KCl electrolyte were used as the anolyte and catholyte, respectively, and cyclically pumped at a flow rate of 20 ml min^−1^. The cell test was performed via NEWARE BTS-5V6A within the voltage window: 0.5–2.2 V at current density of 10 mA cm^−2^. The rate performance of these batteries was investigated with current densities ranging from 10 to 40 mA cm^−2^. The coulombic efficiency (CE), voltage efficiency (VE) and energy efficiency (EE) were calculated^45^:

4 $${\text{CE}}\;(\% ) = \frac{{\int {I_{{\text{d}}} {\text{d}}t} }}{{\int {I_{{\text{c}}} {\text{d}}t} }} \times 100$$

5 $${\text{EE}}\;(\% ) = \frac{{\int {V_{{\text{d}}} I_{{\text{d}}} {\text{d}}t} }}{{\int {V_{{\text{c}}} I_{{\text{c}}} {\text{d}}t} }} \times 100$$

6 $${\text{VE}}\;(\% ) = \frac{{{\text{EE}}}}{{{\text{CE}}}} \times 100$$

where *I*_c_ and *I*_d_ are current of charge and discharge, and *V*_c_ and *V*_d_ are voltage of charge and discharge.

## Results and discussion

### Compressed composite CF characterization

**Figure 2:**
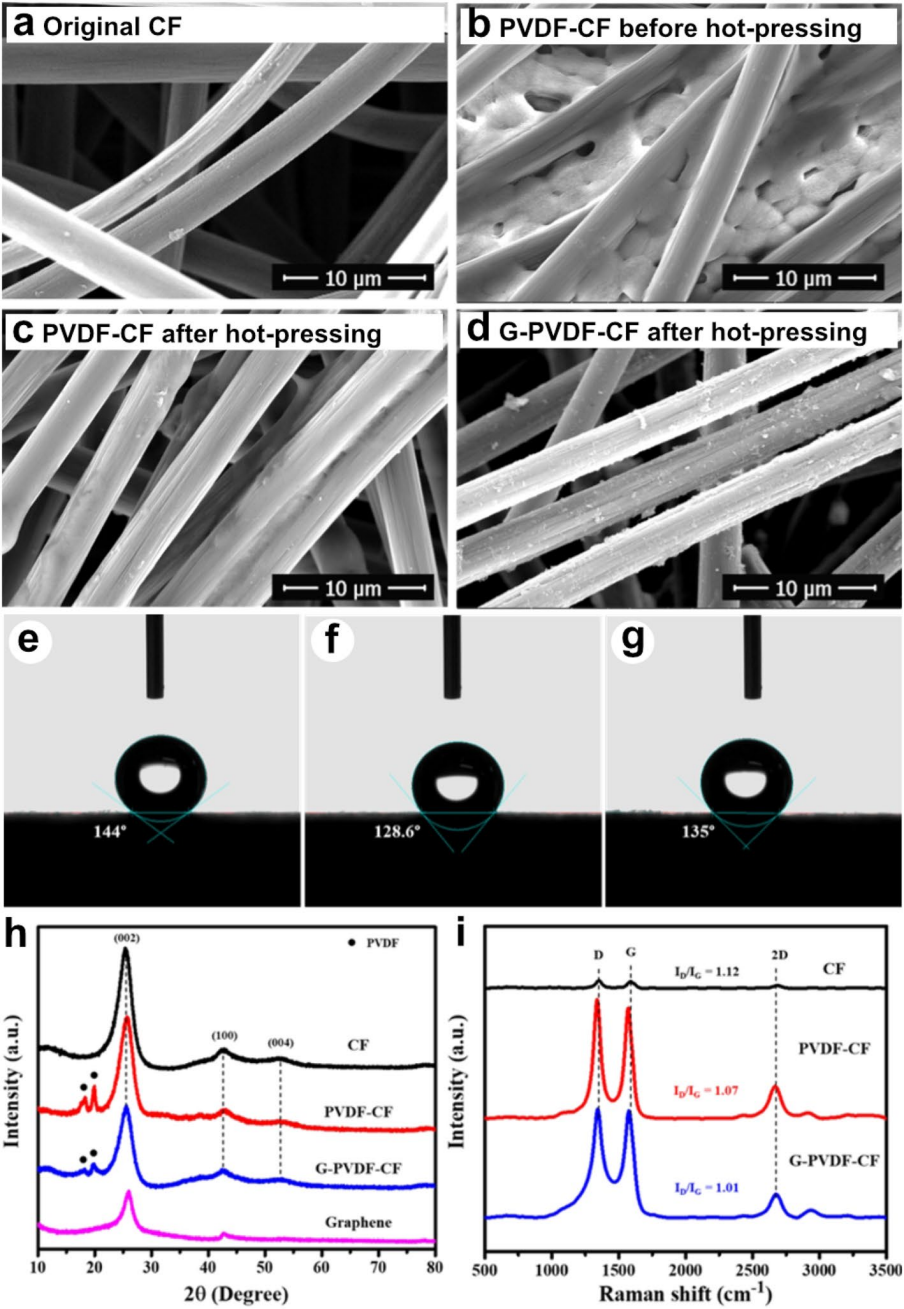
Morphology change during fabrication of compressed composite electrodes: (**a**) original CF, (**b**) PVDF-CF before hot-pressing, (**c**) PVDF-CF after hot-pressing, and (**d**) G-PVDF-CF after hot-pressing; contact angle images with water droplets: (**e**) CF, (**f**) PVDF-CF and (**g**) G-PVDF-CF electrodes; (**h**) XRD patterns, and (**i**) Raman spectra of the CF, PVDF-CF and G-PVDF-CF samples.

In Fig. 2a–c, the morphology changes that occur during the fabrication of a compressed composite CF are illustrated. The original CF contains fibers which each has approximately 8 µm diameter. In Fig. 2a, each carbon fiber is seen to have a relatively smooth surface but having some flaws. In Fig. 2b, PVDF is loaded into the inner space of the CF prior to hot pressing. Figure 2c reveals that after compression the PVDF covers the surface of the carbon fibers linking them together, resulting in better contact with each other. In Fig. 2d, the samples having the addition of graphene were found to be covered with a mass of graphene nanoflakes.

In Fig. 2e–g, the contact angle images of the original CF, PVDF-CF and G-PVDF-CF with water droplets are shown. In Fig. 2e, the CF electrode has a contact angle of 144° after dropping the water onto its surface. In Fig. 2f,g, the PVDF-CF and G-PVDF-CF electrodes reveal lower water contact angles of 128.6° and 135°, respectively, indicating better wettability of the modified electrodes. Such outcomes can affect the composite CF electrodes, leading to better electrochemical performance^46^.

In Fig. 2h, the XRD patterns of the CF, PVDF-CF, G-PVDF-CF electrodes and graphene powder are demonstrated. All XRD patterns show the characteristic reflection peaks of graphite (JCPDS 75-1621) at 2θ of 25.6°, 43.0° and 53.0°, corresponding to the (002), (100), (004) crystal planes, respectively. However, the intensity of the characteristic peaks in the graphitic domain of the composite CF decreased slightly, providing evidence of the incorporation of PVDF. As regards the modified electrodes, the XRD patterns signify the diffraction peaks of PVDF (JCPDS 42-1650) at 2θ of 18.3° and 19.9°. In contrast, the broader peak observed in the G-PVDF-CF sample confirms the existence of graphene on the CF surface, which is consistent with the amorphous nature of graphene.

In Fig. 2i, Raman spectroscopy was used to study the structural changes of the electrode before and after modification. The Raman spectra of each sample show three distinct peaks. Two intense bands, D and G, are located at 1350 and 1580 cm^−1^. Band D is attributed to the disordered graphite structure due to sp^3^ carbon bonds. Band G is a characteristic peak of sp^2^ carbon in the graphitic structure. Band 2D, located at 2690 cm^−1^, is a combination of bands D and G and is also a characteristic band of the graphitic carbon^47,48^. As can be seen, the spectrum of the original CF is relatively weak, but it sharply increases after modification, indicating that the structure of the carbon fiber has changed. The relative intensity of bands D and G i.e. (I_D_/I_G_) represents the degree of graphitization. The I_D_/I_G_ ratio of CF, PVDF-CF and G-PVDF-CF are 1.12, 1.07 and 1.01, respectively, indicating that these values decrease after hot-compressing because the felt compression provides a more ordered structure. Moreover, the introduction of graphene confirms that the G-PVDF-CF electrode has the lowest I_D_/I_G_ ratio among all samples, suggesting the strongest of graphitization characteristic.

In term of surface area, as seen in Fig. S1f, the ESCA of the CF, PVDF-CF and G-PVDF-CF are 337.0, 252.5 and 313.5 cm^2^, respectively. Such a result indicates that the addition of PVDF decreases the overall active area of the CF. However, with the addition of graphene, together with PVDF, the overall active area is almost the same as raw CF; this is due to the additional area from the graphene.

### Electrochemical evaluations

**Figure 3:**
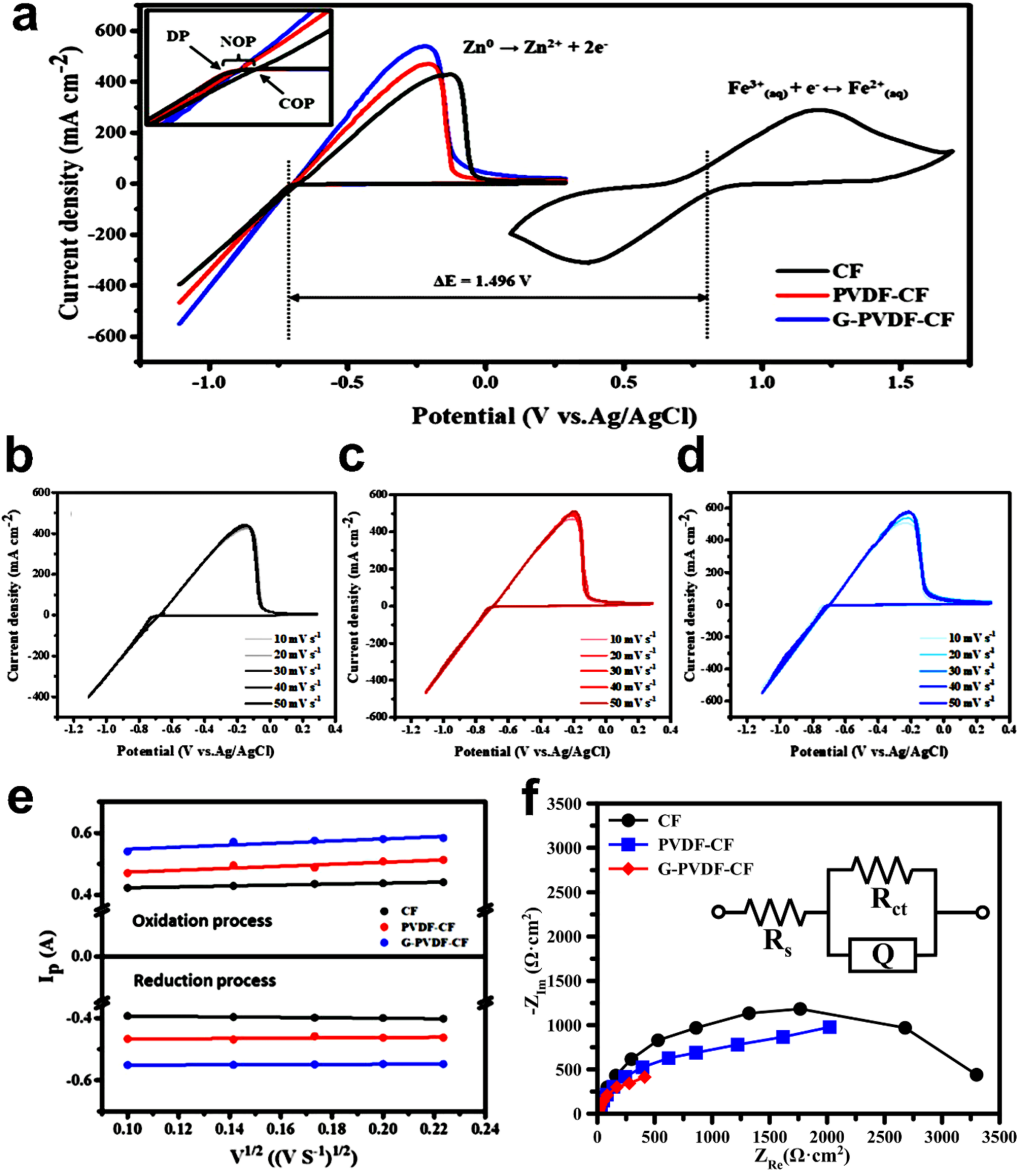
(**a**) cyclic voltammetry of the positive and negative half cells used in this study at a scan rate of 10 mV s^−1^; cyclic voltammograms of (**b**) CF, (**c**) PVDF-CF and (**d**) G-PVDF-CF electrodes at a different scan rate in 0.5 M ZnCl_2_ in 3 M KCl electrolyte; (**e**) anodic and cathodic peak currents versus the square root of the scan rate, and (**f**) Nyquist plots recorded on different electrodes using frequency range of 10 mHz to 1000 kHz and AC amplitude of 10 mV.

In Fig. 3a, the CV curves of zinc and iron redox reactions for the different electrodes acquired at a scanning rate of 10 mV s^−1^ are shown. In the negative half-cell, it is seen that zinc reduction started during the scan in the negative direction. Once Zn started to deposit on the substrate, the corresponding potential is the deposition potential (DP). In an opposite scan, the potential located at the cross point of the current loop is a process for zinc nucleation and growth, which is called the crossover potential (COP). In Fig. 3a (inset), NOP is defined as the potential difference between the crossover point and the deposition point. In Table 1, a comparison of the performances extracted from the CV results for each electrode was summarized. The initial DPs of the modified CFs did not differ from the original one, however, the deposition currents of modified samples were found to be higher than that of the raw CF: this indicates to the improved Zn^2+^/Zn reaction kinetics by the modification. Moreover, smaller NOPs were obtained, which are key parameters for determining the deposition quality from polarization of nucleation. It is seen that the G-PVDF-CF electrode had the smallest value of NOP, suggesting that the deposition on this electrode was more uniform and more efficient. During re-oxidation of the deposited zinc, it is significant that an oxidation peak appeared and the current for the modified electrode was obviously higher than CF, thereby shifting towards negative potential. Thus, the G-PVDF-CF electrode is more favorable compared to the other electrodes for electrochemical activity of the Zn^2+^/Zn redox reactions. Meanwhile, the Fe^3+^/Fe^2+^ redox reaction occurred on the positive potential of 0.784 V vs Ag/AgCl. Hence, the potential difference between the anode and cathode half-cell reactions reached ~ 1.496 V, according to the redox potentials of the Zn–Fe active species.

**Table 1:** CV comparing the electrochemical performance of CFs.

Sample	DP (mV)	COP (mV)	NOP (mV)	Current density at vertex potential (mA/cm^2^)	Anodic limit potential (mV)	Anodic limit current density (mA/cm^2^)
CF	− 713	− 676	37	396	− 130	429
PVDF-CF	− 713	− 696	17	467	− 203	470
G-PVDF-CF	− 712	− 698	14	551	− 217	540

As shown in Fig. 3b–d, the CV curves for the original and modified electrodes used in the present work recorded at various sweep rates ranged from 10 to 50 mV s^−1^ for 0.5 M ZnCl_2_ in the 3 M KCl electrolyte. In Fig. 3e, the peak current density was plotted and linearly fitted via the square root of the scan rate using the Randles–Sevcik equation, indicating a diffusion-controlled reaction process. In Fig. 3f, the EIS results of three samples are shown. Nyquist plots were fitted with an equivalent circuit model (EQCM). The used EQCM consists of the solution resistance (R_s_), the charge transfer resistance (R_ct_) between the electrode/electrolyte at the interface, and constant phase element (CPE), reflecting the double layer capacitance of the electrode/electrolyte interface. In Table 2, the fitting data are listed. Results show that the R_s_ and R_ct_ values of the G-PVDF-CF electrode denote the lowest values among the electrodes, corresponding to 0.96 and 1.10 × 10^3^ Ω, respectively. It is noted that the R_s_ and R_ct_ values decrease in the sequence of CF > PVDF-CF > G-PVDF-CF, suggesting that the modified electrodes can improve the electrochemical activity of the Zn^2+^/Zn redox reaction. Moreover, the modified electrodes exhibited greater CPE values than that of the original CF: this indicates to the improved Zn^2+^/Zn transport and activeness of the electrode surface on Zn^2+^/Zn reaction for the PVDF-CF and G-PVDF-CF samples. These results are well in lined with the CV results. Interestingly, in spite of the lower active area, as seen in ESCA result, the modified CFs still provide better performance than that of raw CF. Such an outcome reflects the fact that the modification using PVDF and graphene one of the effective approaches to improve the surface activity of the CF.

**Table 2:** EIS fitting data of CF, PVDF-CF, and G-PVDF-CF electrodes obtained from Fig. 3f.

Sample	R_s_ (Ω)	R_ct_ (× 10^3^) (Ω)	CPE	
			Y_0_ (10^–3^)	n
CF	1.47	3.72	0.74	0.78
PVDF-CF	1.20	2.06	1.97	0.84
G-PVDF-CF	0.96	1.10	6.50	0.85

**Figure 4:**
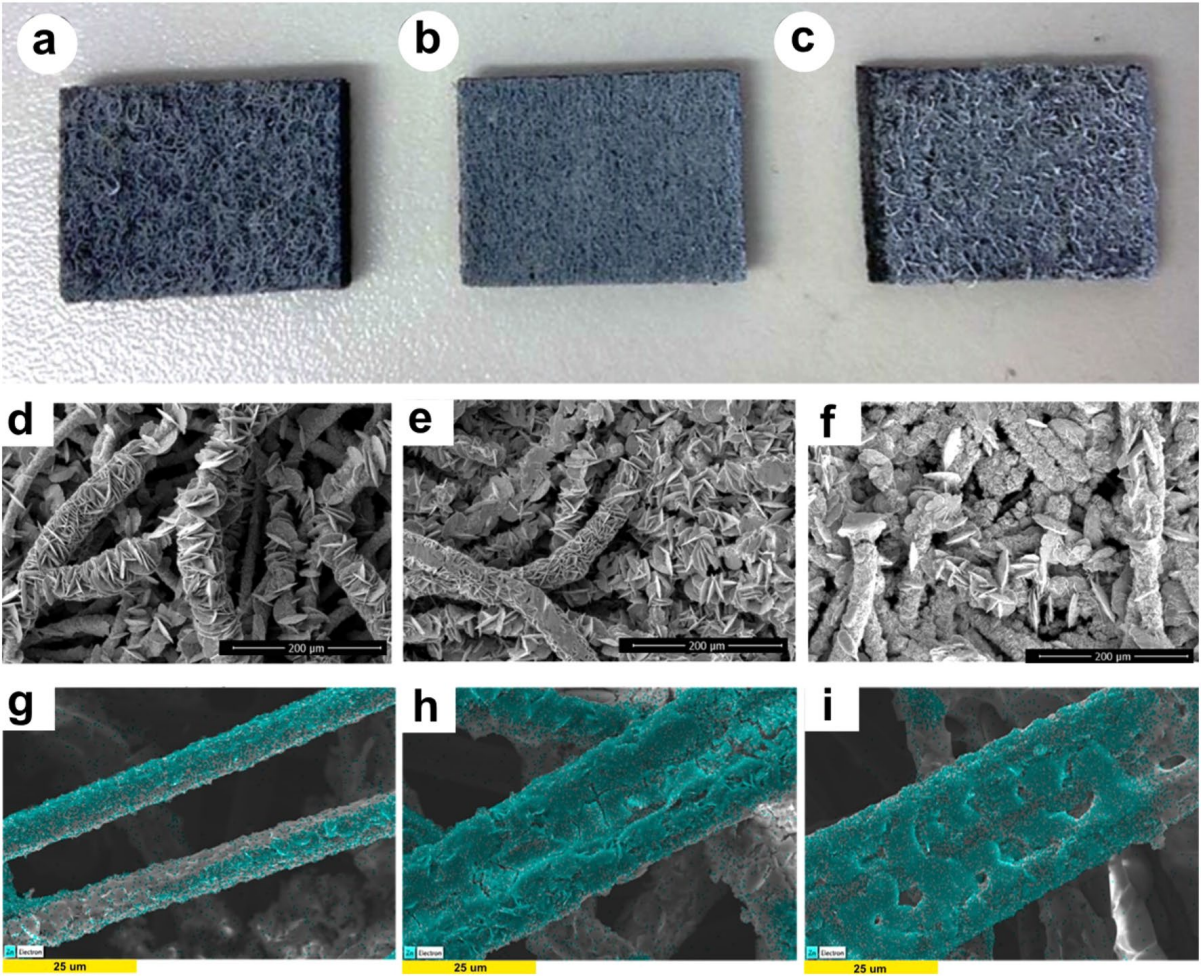
Photos of the as-prepared electrodes after zinc electrodeposition: (**a**) CF, (**b**) PVDF-CF and (**c**) G-PVDF-CF; FESEM images: (**d**) CF, (**e**) PVDF-CF and (**f**) G-PVDF-CF electrodes after electrodeposition of Zn; energy dispersive X-ray (EDX) mapping analysis of (**g**) CF, (**h**) PVDF-CF and (**i**) G-PVDF-CF electrodes after electrodeposition of Zn.

Different surfaces of Zn electrodeposited on the CF, PVDF-CF and G-PVDF-CF electrodes can be observed from the photographs shown in Fig. 4a–c. To gain an insight into the morphologies of each electrode, FESEM images are examined. The morphology of the zinc electrodeposits on the original CF reveals a hexagonal-like flake structure (Fig. 4d). As for the composite CFs, a more compact deposition layer of Zn is observed and the addition of graphene results in a decrease in the grain size of Zn (Fig. 4e,f). This outcome implies that the connection between carbon fiber offers active sites, facilitating adsorption of zinc ions, as evidenced by the EDX mapping in Fig. 4g–i.

**Figure 5:**
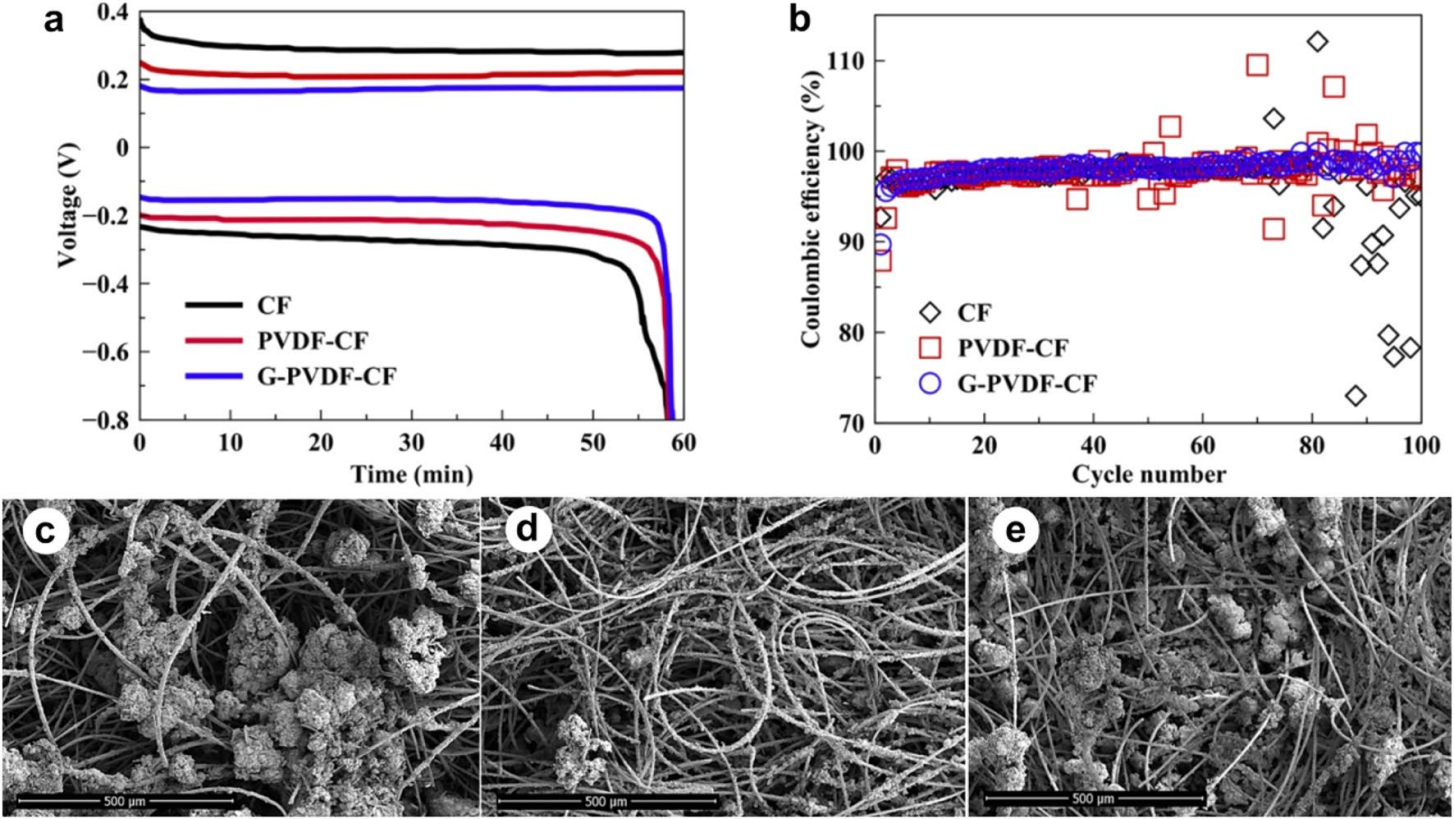
(**a**) Voltage profiles recorded during Zn plating/stripping at cycle 20th using 10 mAh cm^−2^ areal capacity, and 10 mA cm^−2^ current density and (**b**) coulombic efficiency of Zn plating/stripping vs. cycle number; FESEM images of (**c**) CF, (**d**) PVDF-CF and (**e**) G-PVDF-CF electrodes after 50 cycles of Zn plating/stripping test.

The plating/stripping performance of the compressed composite electrode on the deposition of Zn was investigated. For charging, Zn was plated on the electrode at a constant current density of 10 mA cm^−2^, which lasted for an hour. Then, for discharging, Zn was stripped with the same current density until the voltage reached cut-off at 0.8 V. As can be seen in Fig. 5a, modification of the electrodes results in minimizing the size of overpotential both during charging and discharging. Such outcomes reflect that modified CFs display better Zn compatibility which results in lowering the energy consumption upon the Zn deposition/dissolution. Furthermore, the cycling performance of Zn plating/stripping was used to further evaluate its reversibility (Fig. 5b). Every electrode demonstrated coulombic efficiency (CE) of around 90% at the 1st cycle. Then, the CE of all samples increased and reached the stable plateau at around 5th cycle yielding the average CE of 97–98%. For the CF sample and PVDF-CF sample, after many cycles have pass, the fluctuation of CE values was observed. Such outcomes may occur as a consequence of the hydrogen gas production within the cell which can disconnect the contact between electrode and electrolyte, thus, result in the interrupting charging and discharging processes. On the other hand, the graphene contained sample (G-PVDF-CF) exhibit a smooth trend from the start until the end of the test: this may reflect that graphene could reduce the hydrogen evolution.

**Figure 6:**
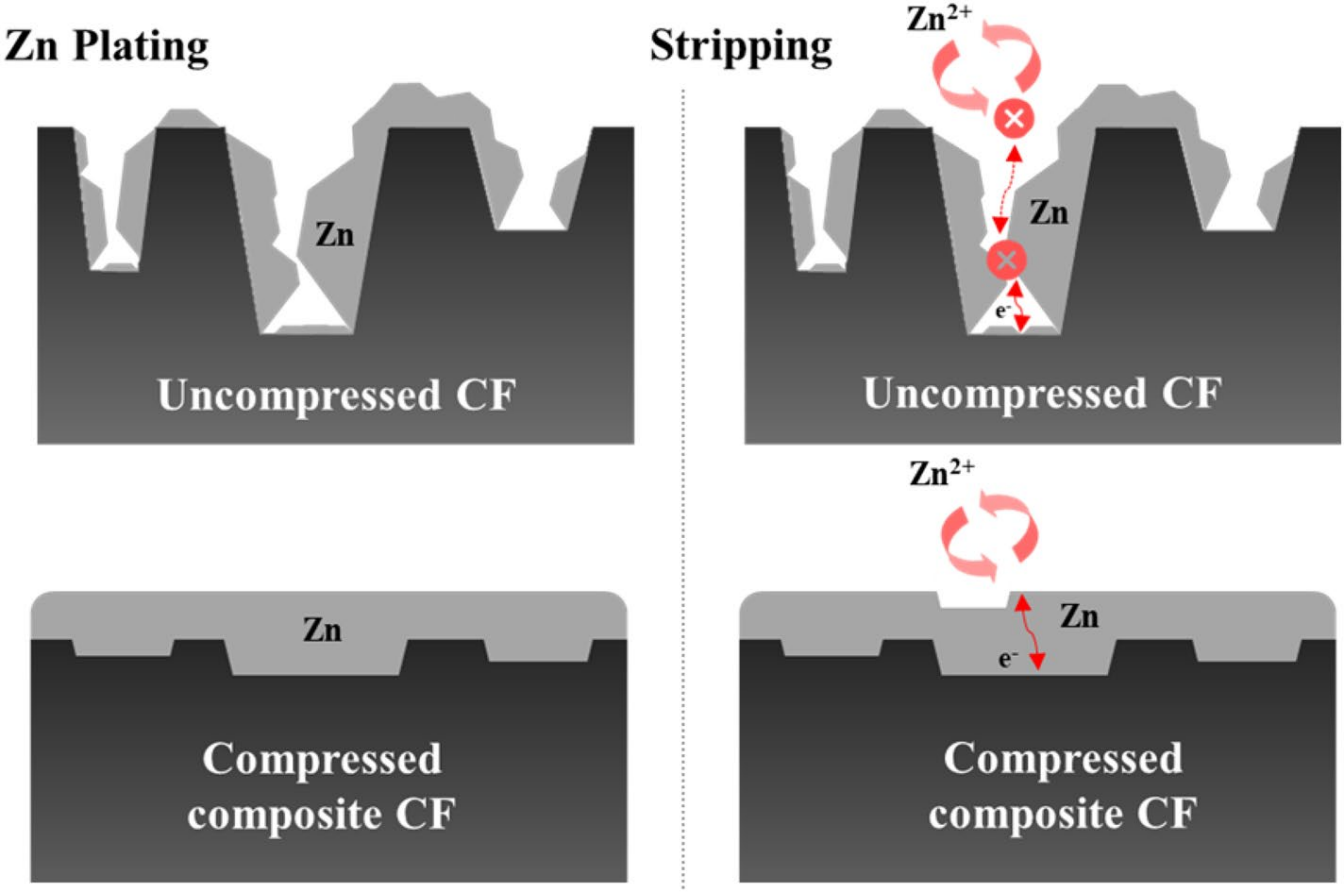
Schema of Zn plating/stripping on uncompressed CF and compressed composited CF electrodes.

FESEM analysis was employed to characterize the electrodeposit morphology again, after the charging process. The CF electrode with a porous Zn deposit exhibited an aggressive agglomeration and non-planar morphology (Fig. 5c). As a consequence, ion transport became sluggish and electric field became nonuniform, resulting in detached fragments and low plating/stripping efficiency. Meanwhile, the compressed composite electrodes viz. PVDF-CF and G-PVDF-CF having a compact deposition layer are seen to avoid the severe Zn agglomeration (Fig. 5d,e). Such an outcome occurred because the compact Zn deposition layer sustained the ion transport and uniform electric field. It is evident that electrochemical dissolution can occur only on the surface of the electrode. It is also noted that surfaces of the G-PVDF-CF electrode were not smooth. In Fig. 6, a graphical illustration of ion transport during Zn plating/stripping on the uncompressed CF and compressed composited CF electrodes is depicted.

**Figure 7:**
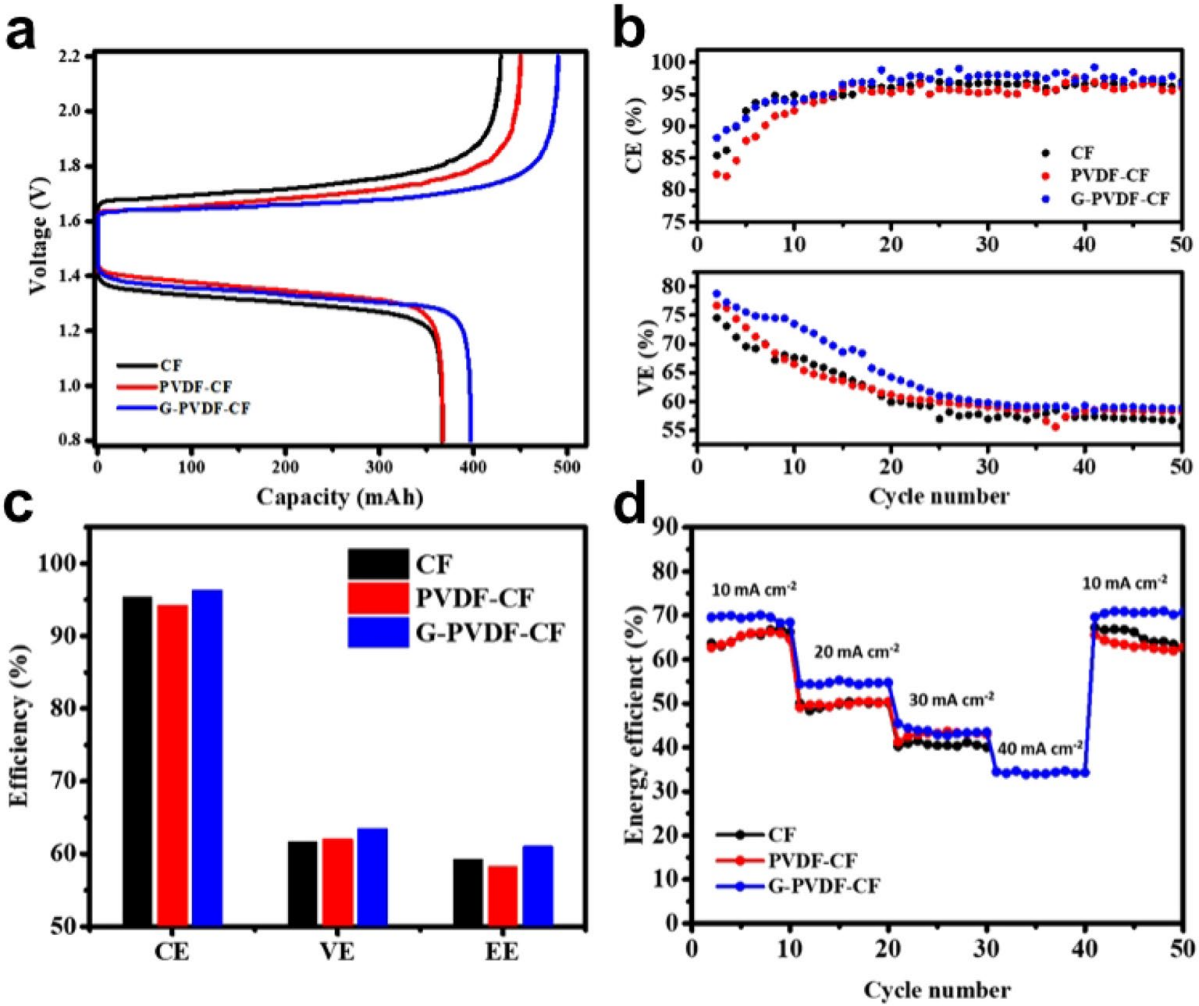
Electrochemical performance of ZFB with CF, PVDF-CF, and G-PVDF-CF electrodes: (**a**) charge–discharge capacity curves at current density: 10 mA cm^−2^, (**b**) results of CE and VE of ZFB, (**c**) average efficiency during 50 cycles, and (**d**) energy efficiency changes as a function of cycle numbers under various current densities: 10–40 mA cm^−2^.

The practical application of CF, PVDF-CF and G-PVDF-CF as negative electrodes used in Zn–Fe flow batteries was determined. Figure 7a demonstrates both charge and discharge curves for the second cycle at a constant current density of 10 mA cm^−2^. The curves show that the charge and discharge voltages of the cell with the modified CF electrodes have lower overpotential than the original CF, implying that felt compression can reduce electrode polarization, resulting in greater capacity. The maximum discharge capacity of 396 mAh for the G-PVDF-CF electrode was obtained over the same operation voltage window (0.8–2.2 V), which was 7.6% higher than the CF electrode. It is acknowledged that a PVDF-coating layer can influence conductivity in batteries. As a result, the discharge capacity of 366 mAh for the PVDF-CF electrode was similar to that of the CF electrode. In Fig. 7b, both coulombic efficiency, and voltage efficiency of each electrode over 50 cycles was compared. In Fig. 7c, the average efficiency of ZFB is presented. In accordance with the charge–discharge curves, the batteries with the G-PVDF-CF electrode provided better performances compared with those of a ZFB with CF and PVDF-CF electrodes at a current density of 10 mA cm^−2^. The CE, VE and EE values of G-PVDF-CF are 96.4%, 63.4% and 61%, respectively. The batteries based on the PVDF-CF electrode are observed to have a slightly higher VE than the original CF, verifying lower electrode polarization during operation. Figure 7d shows the energy efficiency of ZFBs assembled with each electrode at various current densities, ranging from 10 to 40 mA cm^−2^.

Because of higher polarization overpotential, EE decreases dramatically as current density increases. The ZFB single cell assembled with the PVDF-CF revealed a similar EE tendency as the CF electrode being unable to complete the charge–discharge curves at a current density of 40 mA cm^−2^ due to excessive electrochemical overpotential. Besides, it is noted that a ZFB with G-PVDF-CF electrode demonstrated EE at a current density of 40 mA cm^−2^, and completed its operation under the conditions investigated. Thus, the compressed composite G-PVDF-CF electrode exhibited the best performance in terms of capacity and stability achieving improvements in wettability, deposition efficiency as well as mechanical property. Herein, as observed in Table 3, the improvements and performances recorded demonstrate the use of the negative electrode, indicating the immense potential for the G-PVDF-CF electrode and its applications in ZFBs.

**Table 3:** Performance comparison of Zn–Fe redox flow batteries and this work.

Anode	Cathode	Membrane	Anolyte	Catholyte	Areal capacity (mAh cm^−2^)	Volumetric capacity (Ah L^−1^)	References
Zn foil	CF	HZ115	1 M ZnSO_4_	1 M FeCl_2_ + 1.5 M H_2_SO_4_	10	2.4	^49^
			1 M ZnSO_4_ + 1.5 M HAc + 1.5 M NaAc		97	23.5	
CF	CF	PBI	0.4 M ZnBr_2_ in 2 M KCl	0.8 M FeCl_2_ in 2 M KCl	20.6	16.5	^20^
				0.8 M FeCl_2_ + 1.6 glycine in 2 M KCl	22.5	18	
Zn foil	CF	Nafion115	0.1 M ZnCl_2_ in 2 M NaCl	0.1 M Fe(bpy)_3_Cl_2_ in 2 M NaCl	-	2.8	^50^
CF	CF	Nafion117	0.1 M ZnBr_2_ in 2 M KCl	0.2 M FeCl_2_ + 0.4 M glycine in 2 M KCl	9.26	3.12	^22^
		SPEEK-K			10.8	3.65	
CF	CF	Nafion117	0.5 M ZnCl_2_ in 3 M KCl	0.25 M FeCl_3_ + 0.25 M FeCl_2_ in 2 M KCl	36.6	8.1	This work
G-PVDF-CF	CF				39.6	8.8	

## Conclusions

In this paper, a G-PVDF-CF electrode was synthesized by immersing CF in a PVDF solution containing graphene powder under ultrasonic sonicating. According to cyclic voltammetry results, the G-PVDF-CF electrode had the highest deposition currents and the lowest NOP, resulting in a more uniform deposition of zinc on its surface, as evidenced by FESEM images of the compact zinc deposition layer. Moreover, the plating/stripping efficiency of zinc was tested and confirmed the higher performance of plating/stripping reversibility for modified electrodes with no severe zinc agglomeration. Furthermore, G-PVDF-CF exhibited the best charge–discharge characteristics at current density of 10 mA cm^−2^ with a discharge capacity of 39.6 mAh cm^−2^ due to lower overpotential than the original CF. During 50 cycles, the batteries using the G-PVDF-CF electrode demonstrated CE, VE, and EE values of 96.4%, 63.4%, and 61%, respectively, displaying their robustness and rate capability.

## Supplementary Information

Supplementary Figure S1.

## Data Availability

All data generated or analyzed during this study are included in this published article.

## References

[1] Hannan MA (2021). Battery energy-storage system: A review of technologies, optimization objectives, constraints, approaches, and outstanding issues. J. Energy Storage.

[2] Olaru, S., Stoican, F. & Kheawhom, S. in *2021 IEEE AFRICON.*10.1109/AFRICON51333.2021.9571020.

[3] Guerra OJ (2020). The value of seasonal energy storage technologies for the integration of wind and solar power. Energy Environ. Sci..

[4] Abbasi A (2020). Discharge profile of a zinc-air flow battery at various electrolyte flow rates and discharge currents. Sci. Data.

[5] Khezri R (2022). Performance enhancement through parameter optimization for a rechargeable zinc-air flow battery. J. Ind. Eng. Chem..

[6] Poolnapol L (2020). Silver decorated reduced graphene oxide as electrocatalyst for zinc-air batteries. Energies.

[7] Lao-atiman W, Bumroongsri P, Arpornwichanop A, Olaru S, Kheawhom S (2022). Prediction of charge-discharge behavior and state of charge estimation for tri-electrode rechargeable zinc-air flow batteries. J. Energy Storage.

[8] Winsberg J, Hagemann T, Janoschka T, Hager MD, Schubert US (2017). Redox-flow batteries: From metals to organic redox-active materials. Angew. Chem. Int. Ed..

[9] Khezri R (2022). Stabilizing zinc anodes for different configurations of rechargeable zinc-air batteries. Chem. Eng. J..

[10] Zhang J (2018). An all-aqueous redox flow battery with unprecedented energy density. Energy Environ. Sci..

[11] Lao-atiman W, Olaru S, Arpornwichanop A, Kheawhom S (2019). Discharge performance and dynamic behavior of refuellable zinc-air battery. Sci. Data.

[12] Shi Y (2019). Recent development of membrane for vanadium redox flow battery applications: A review. Appl. Energy.

[13] Gencten M, Sahin Y (2020). A critical review on progress of the electrode materials of vanadium redox flow battery. Int. J. Energy Res..

[14] Yuan X-Z (2019). A review of all-vanadium redox flow battery durability: Degradation mechanisms and mitigation strategies. Int. J. Energy Res..

[15] Tangthuam P (2020). Carboxymethyl cellulose-based polyelectrolyte as cationic exchange membrane for zinc–iodine batteries. Heliyon.

[16] Ulaganathan M, Suresh S, Mariyappan K, Periasamy P, Pitchai R (2019). New zinc–vanadium (Zn–V) hybrid redox flow battery: High-voltage and energy-efficient advanced energy storage system. ACS Sustain. Chem. Eng..

[17] Zeng Y, Yang Z, Lu F, Xie Y (2019). A novel tin-bromine redox flow battery for large-scale energy storage. Appl. Energy.

[18] Zai J (2020). Sandwiched Cu7S4@graphite felt electrode for high performance aqueous polysulfide/iodide redox flow batteries: Enhanced cycling stability and electrocatalytic dynamics of polysulfides. Mater. Chem. Phys..

[19] Zhang H, Sun C (2021). Cost-effective iron-based aqueous redox flow batteries for large-scale energy storage application: A review. J. Power Sources.

[20] Xie C, Duan Y, Xu W, Zhang H, Li X (2017). A low-cost neutral zinc–iron flow battery with high energy density for stationary energy storage. Angew. Chem. Int. Ed..

[21] Yuan Z, Duan Y, Liu T, Zhang H, Li X (2018). Toward a low-cost alkaline zinc–iron flow battery with a polybenzimidazole custom membrane for stationary energy storage. iScience.

[22] Chang S (2019). A low-cost SPEEK-K type membrane for neutral aqueous zinc–iron redox flow battery. Surf. Coat. Technol..

[23] Selverston S, Savinell RF, Wainright JS (2017). Zinc–iron flow batteries with common electrolyte. J. Electrochem. Soc..

[24] Wang Y, Chang Z, Li J, Li R, Huang F (2018). Zinc ferrum energy storage chemistries with high efficiency and long cycling life. J. Mater. Chem. A.

[25] Jiang HR, Wu MC, Ren YX, Shyy W, Zhao TS (2018). Towards a uniform distribution of zinc in the negative electrode for zinc bromine flow batteries. Appl. Energy.

[26] Shimizu M, Hirahara K, Arai S (2019). Morphology control of zinc electrodeposition by surfactant addition for alkaline-based rechargeable batteries. Phys. Chem. Chem. Phys..

[27] Wang S (2021). A highly reversible zinc deposition for flow batteries regulated by critical concentration induced nucleation. Energy Environ. Sci..

[28] Jabbari V, Foroozan T, Shahbazian-Yassar R (2021). Dendritic Zn deposition in zinc-metal batteries and mitigation strategies. Adv. Energy Sustain. Res..

[29] Liu T, Li X, Nie H, Xu C, Zhang H (2015). Investigation on the effect of catalyst on the electrochemical performance of carbon felt and graphite felt for vanadium flow batteries. J. Power Sources.

[30] Zhang H, Chen N, Sun C, Luo X (2020). Investigations on physicochemical properties and electrochemical performance of graphite felt and carbon felt for iron-chromium redox flow battery. Int. J. Energy Res..

[31] Averbukh M, Lugovskoy S (2019). Theoretical description of carbon felt electrical properties affected by compression. Appl. Sci..

[32] Banerjee R (2019). Carbon felt electrodes for redox flow battery: Impact of compression on transport properties. J. Energy Storage.

[33] Emmel D (2020). Understanding the impact of compression on the active area of carbon felt electrodes for redox flow batteries. ACS Appl. Energy Mater..

[34] Wang Q, Qu ZG, Jiang ZY, Yang WW (2018). Experimental study on the performance of a vanadium redox flow battery with non-uniformly compressed carbon felt electrode. Appl. Energy.

[35] Chang T-C, Zhang J-P, Fuh Y-K (2014). Electrical, mechanical and morphological properties of compressed carbon felt electrodes in vanadium redox flow battery. J. Power Sources.

[36] Park S-K (2014). The influence of compressed carbon felt electrodes on the performance of a vanadium redox flow battery. Electrochim. Acta.

[37] Brown LD (2016). The effect of felt compression on the performance and pressure drop of all-vanadium redox flow batteries. J. Energy Storage.

[38] Liu Z, Wang B, Yu L (2018). Preparation and surface modification of PVDF-carbon felt composite bipolar plates for vanadium flow battery. J. Energy Chem..

[39] Hieu LT, So S, Kim IT, Hur J (2021). Zn anode with flexible β-PVDF coating for aqueous Zn-ion batteries with long cycle life. Chem. Eng. J..

[40] Opar DO, Nankya R, Lee J, Jung H (2020). Three-dimensional mesoporous graphene-modified carbon felt for high-performance vanadium redox flow batteries. Electrochim. Acta.

[41] Li W (2016). Graphene-nanowall-decorated carbon felt with excellent electrochemical activity toward VO2+/VO2+ couple for all vanadium redox flow battery. Adv. Sci..

[42] Etesami M (2022). 3D carbon nanotubes-graphene hybrids for energy conversion and storage applications. Chem. Eng. J..

[43] Xiong J (2018). Mechanical modelling and simulation analyses of stress distribution and material failure for vanadium redox flow battery. J. Energy Storage.

[44] Abdulla J (2021). Elimination of zinc dendrites by graphene oxide electrolyte additive for zinc-ion batteries. ACS Appl. Energy Mater..

[45] Xi J (2015). Effect of degree of sulfonation and casting solvent on sulfonated poly(ether ether ketone) membrane for vanadium redox flow battery. J. Power Sources.

[46] Jeon DH (2019). Wettability in electrodes and its impact on the performance of lithium-ion batteries. Energy Storage Mater..

[47] Lim TH, Yeo SY (2017). Investigation of the degradation of pitch-based carbon fibers properties upon insufficient or excess thermal treatment. Sci. Rep..

[48] Casimero C, Hegarty C, McGlynn RJ, Davis J (2020). Ultrasonic exfoliation of carbon fiber: Electroanalytical perspectives. J. Appl. Electrochem..

[49] Xie Z (2016). High performance of zinc-ferrum redox flow battery with Ac-/HAc buffer solution. J. Energy Chem..

[50] Xie Z, Wei L, Zhong S (2020). An aqueous ZnCl_2_/Fe(bpy)3Cl_2_ flow battery with mild electrolyte. Front. Mater. Sci..

