# Author Correction: Compressed composite carbon felt as a negative electrode for a zinc–iron flow battery

**DOI:** 10.1038/s41598-023-32392-z

**Published:** 2023-04-04

**Authors:** Janenipa Saupsor, Jinnawat Sangsawang, Wathanyu Kao-ian, Falko Mahlendorf, Ahmad Azmin Mohamad, Rongrong Cheacharoen, Soorathep Kheawhom, Anongnat Somwangthanaroj

**Affiliations:** 1 Department of Chemical Engineering, Faculty of Engineering, Chulalongkorn University, Bangkok, 10330 Thailand; 2 Department of Energy Technology, University Duisburg-Essen, 47057 Duisburg, Germany; 3 School of Materials and Mineral Resources Engineering, Universiti Sains Malaysia, 14300 Nibong Tebal, Pulau Pinang Malaysia; 4 Metallurgy and Materials Science Research Institute, Chulalongkorn University, Bangkok, 10330 Thailand; 5 Center of Excellence on Advanced Materials for Energy Storage, Chulalongkorn University, Bangkok, 10330 Thailand; 6 Bio-Circular-Green-Economy Technology and Engineering Center (BCGeTEC), Faculty of Engineering, Chulalongkorn University, Bangkok, 10330 Thailand

Correction to: *Scientific Reports* 10.1038/s41598-022-25763-5, published online 07 December 2022

The original version of this Article contained an error in the Acknowledgements section, where the grant number of the Program Management Unit for Human Resources and Institutional Development, Research and Innovation was incorrect.

“The Program Management Unit for Human Resources and Institutional Development, Research and Innovation (B16F630071), National Science and Technology Development Agency through the Southeast Asia-Europe Joint Funding Scheme for Research and Innovation (FDA-CO-2563-11897-TH), and the Energy Storage Cluster, Chulalongkorn University are acknowledged.”

now reads:

“The Program Management Unit for Human Resources and Institutional Development, Research and Innovation (B16F640166), National Science and Technology Development Agency through the Southeast Asia-Europe Joint Funding Scheme for Research and Innovation (FDA-CO-2563-11897-TH), and the Energy Storage Cluster, Chulalongkorn University are acknowledged.”

The original article has been corrected.

