# Supplementary material for: Compressed composite carbon felt as a negative electrode for a zinc–iron flow battery
Source: Sci Rep. 2022 Dec 7;12:21156. doi: 10.1038/s41598-022-25763-5 (PMC9729305; doi:10.1038/s41598-022-25763-5)
Supplement: Supplementary file 1 — Supplementary Figure S1. [file 41598_2022_25763_MOESM1_ESM.docx]

**Supporting Information**

**Compressed composite carbon felt as a negative electrode for a zinc-iron flow battery**

Janenipa Saupsor^1^, Jinnawat Sangsawang^1^, Wathanyu Kao-ian^1^, Falko Mahlendorf^2^, Ahmad Azmin Mohamad^3^, Rongrong Cheacharoen^4^, Soorathep Kheawhom^1,5,6,*^, Anongnat Somwangthanaroj^1,6,**^

*^1^ Department of Chemical Engineering, Faculty of Engineering, Chulalongkorn University, Bangkok 10330, Thailand*

*^2^Department of Energy Technology, University Duisburg-Essen, Duisburg 47057, Germany*

*^3^School of Materials and Mineral Resources Engineering, Universiti Sains Malaysia, Nibong Tebal, Pulau Pinang 14300, Malaysia*

*^4^Metallurgy and Materials Science Research Institute, Chulalongkorn University, Bangkok 10330, Thailand*

*^5^Center of Excellence on Advanced Materials for Energy Storage,, Chulalongkorn University, Bangkok 10330, Thailand*

*^6^Bio-Circular-Green-economy Technology & Engineering Center (BCGeTEC), Faculty of Engineering, Chulalongkorn University, Bangkok 10330, Thailand*

**Corresponding author: soorathep.k@chula.ac.th*

***Corresponding author: anongnat.s@chula.ac.th*


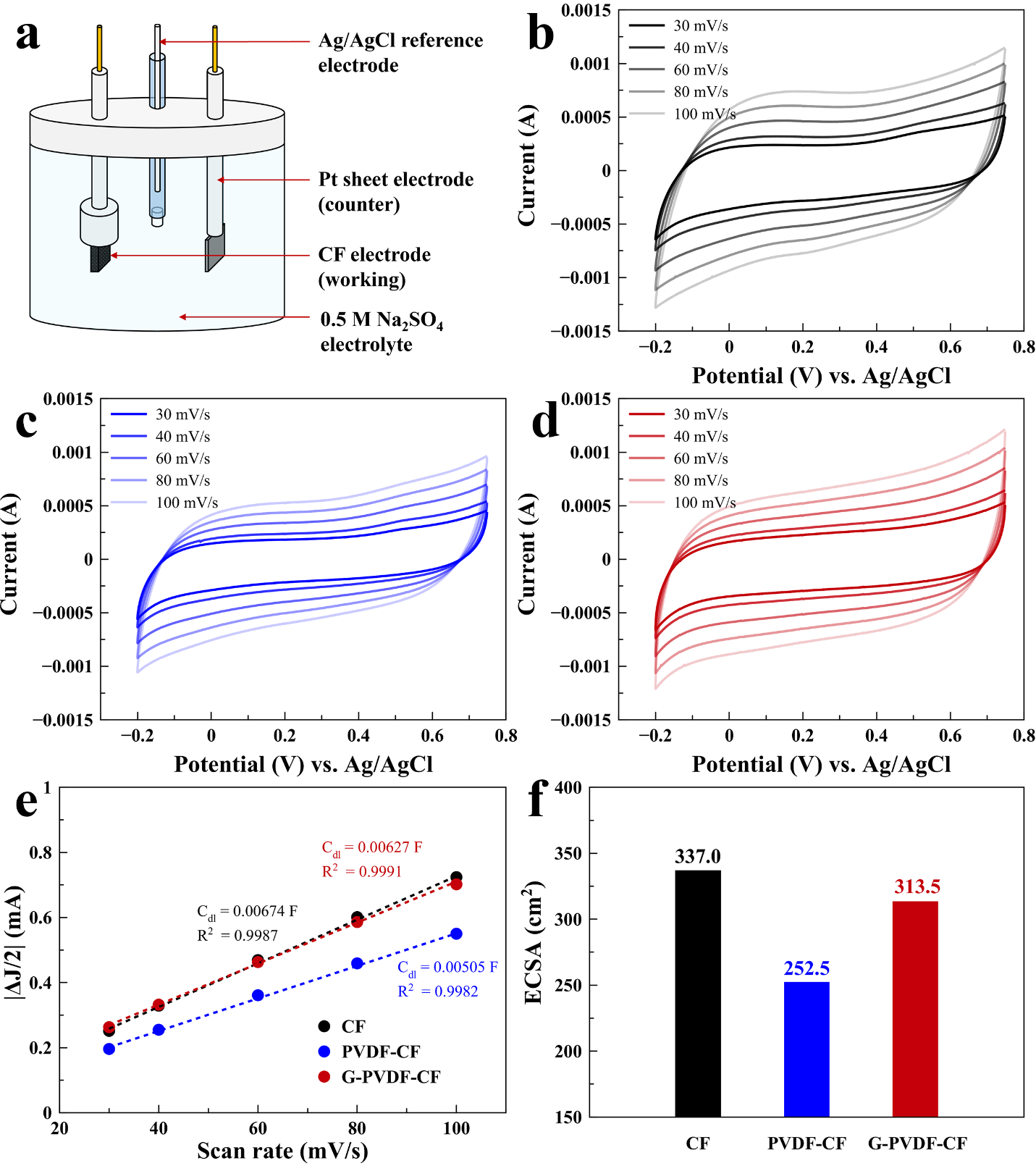


**Figure S1.** (a) Schematic illustrates the cell setup for the ECSA measurement (b)-(d) cyclic voltammograms of the CF sample, the PVDF-CF sample, and the G-PVDF-CF sample, respectively, within potential window of -0.2 V to 0.75 V vs. Ag/AgCl at scan rate 30, 40, 60, 80 and 100 mV/s (e) the linear regression for calculating the capacitance (C_dl_) of the samples (f) ECSA values of the samples having 1x1 cm^2^ and 3 mm thickness
